# Platelet-to-lymphocyte ratio: A new inflammatory marker for the diagnosis of preterm premature rupture of membranes

**DOI:** 10.4274/jtgga.2017.0028

**Published:** 2017-09-01

**Authors:** Erzat Toprak, Murat Bozkurt, Burcu Dinçgez Çakmak, Emel Ebru Özçimen, Musa Silahlı, Ayşe Ender Yumru, Eray Çalışkan

**Affiliations:** 1 Department of Obstetrics and Gynecology, Başkent University School of Medicine, Konya Training and Research Hospital, Konya, Turkey; 2 Department of Obstetrics and Gynecology, Sakarya University School of Medicine, Sakarya, Turkey; 3 Department of Obstetrics and Gynecology, Bursa Yüksek İhtisas Training and Research Hospital, Bursa, Turkey; 4 Department of Neonatalogy, Başkent University School of Medicine, Konya Training and Research Hospital, Konya, Turkey; 5 Department of Obstetrics and Gynecology, Şişli Hamidiye Etfal Training and Research Hospital, İstanbul, Turkey; 6 Department of Obstetrics and Gynecology, Bahçeşehir University School of Medicine, İstanbul, Turkey

**Keywords:** Inflammatory markers, platelet-to-lymphocyte ratio, preterm premature rupture of membranes

## Abstract

**Objective:**

Preterm premature rupture of membranes (PPROM) is closely related with maternal and fetal complications. Therefore, early diagnosis is extremely important to provide maternal and fetal well-being. Many inflammatory markers have been evaluated for their ability to diagnose membrane rupture at early stages. We aimed to investigate the relationship between the platelet-to-lymphocyte ratio (PLR) and preterm premature membrane rupture.

**Material and Methods:**

In this study, 121 pregnant women with PPROM and 96 age-matched pregnant women with spontaneous preterm labor who were admitted to our hospital between January 2014 and December 2015 were enrolled. Demographic data, complete blood cell count results, and neonatal outcomes were recorded.

**Results:**

The neutrophil and platelet counts were higher in the PPROM group (9948.4±3393.2 vs. 7466.1±1698.5/mm^3^ and 244.5±60 vs. 210.6±64.8/mm^3^, respectively, p<0.001). The PLR and neutrophil-to-lymphocyte ratios (NLR) were both significantly higher in the PPROM group (p<0.001). Correlation analysis revealed that the PLR was positively correlated with the NLR (r=0.10, p=0.031). The ability of the PLR to diagnose preterm premature rupture of membranes was evaluated using an ROC curve. The sensitivity and specificity of the PLR was 57.8% and 73.7%, respectively, at a threshold >117.14 (p<0.001).

**Conclusion:**

The PLR might be a cost effective, easy to use, and practical marker for the early diagnosis of PPROM, which can help to determine the appropriate waiting time for delivery and provide maternal and fetal well-being.

## INTRODUCTION

Preterm premature rupture of membranes (PPROM), which is defined as spontaneous rupture of fetal membranes before labor begins before 37 weeks’ gestation, affects approximately 3% of all pregnancies (1). It is closely related with significant maternal and fetal morbidity and mortality. PPROM is one of the most common causes of preterm delivery, and is associated with maternal and neonatal infections (2, 3). The risk of chorioamnionitis is approximately 6-10% and increases to 40% if it prolongs over 24 hours (4). Moreover, neonatal infection risk is two times greater in patients without chorioamnionitis (5). Infection risk increases with PPROM, and neonatal hypoxia and jaundice are also more common in this condition (6). Early diagnosis is very important to provide maternal and fetal well-being because of these serious complications (7). Even though the pathophysiologic mechanism of PPROM has not been clearly defined and is multifactorial; inflammation plays a crucial role in the rupture of membranes (8). The role of inflammation in PPROM has been evaluated in many studies, and a significant association between various inflammatory markers and PPROM has been reported (9, 10, 11). Many inflammatory markers were recently evaluated for their ability to diagnose membrane rupture at early stages.

In chronic inflammatory processes, megakaryocytic series proliferate increasingly and lymphocyte counts tend to decrease due to severe apoptosis. As a consequence, markers obtained from total blood counts such as the platelet-to-lymphocyte ratio (PLR) can be affected in severe chronic inflammatory diseases (12).

PLR is a widely available, effective, and simple marker. It has been proposed as a predictive and prognostic parameter for many kinds of diseases such as cardiovascular diseases and malignancies (13, 14). Also, it has been shown to be related with gestational diabetes mellitus, acute appendicitis, preeclampsia, recurrent pregnancy loss, and preterm labor in pregnant women (15, 16, 17, 18). There are scant data about the relation between PLR and presence of PPROM in the literature. Therefore, we investigated the role of PLR for predicting PPROM at early stages.

## MATERIAL AND METHODS

### Study population and data collection

This is a prospective case-control study, in which 121 pregnant women with PPROM and 96 age- matched pregnant women with spontaneous preterm labor between January 2014 and December 2015 were enrolled. It was conducted at a university-affiliated research and training hospital.

Age, gestational week, gravida, parity, delivery mode, birth weight, APGAR score, neonatal intensive care unit (NICU) admission rate, presence of neonatal sepsis, and development of respiratory distress syndrome (RDS) were recorded from medical records. In addition, results of a complete series of routine laboratory investigations including complete blood cell counts were recorded. Blood samples were taken from all study participants on admission and complete blood counts were analyzed using a Coulter LH 780 Hematology Analyzer (Beckman Coulter Ireland INC, Mervue, Galway, Ireland). The neutrophil-to-lymphocyte ratio (NLR) was calculated by dividing the neutrophil count by the lymphocyte count, and PLR was calculated as the number of platelets divided by the lymphocyte count, both of which were obtained from the same blood samples.

### Inclusion criteria

The inclusion criteria included PPROM diagnosed as defined between 24-37 gestational weeks of pregnancy, and eligible for recording complete blood samples and other clinical perinatal findings.

### Exclusion criteria

We excluded patients with multiple gestations, hematologic disorders, malignancies, hepatic disease, history of autoimmune disease, any inflammatory disease of pregnancy such as gestational diabetes mellitus and preeclampsia, any acute or chronic infectious or inflammatory diseases, pregnancies with fetal chromosomal anomalies, intrauterine growth restriction, any fetal infection, and women who underwent any invasive procedures such as amniocentesis.

### Diagnosis of preterm premature rupture of membranes

PPROM was diagnosed if 1 and one of the other following were present; 1) all patients were asked for risk factors and any fluid leakage before 37 weeks’ gestation and regular uterine contractions, 2) examination in dorsolithotomy position with a sterile speculum to verify the pooling of amniotic fluid in the fornices or active flowing of amniotic fluid from the cervix, 3) positive nitrazine test, 4) when necessary, confirming the presence of insulin-like growth factor binding proteins (ACTIM PROM test; MedixBiochemica, Kauniainen, Finland) in the vaginal fluid.

All participants gave informed consent and the local ethics committee approved the study.

### Statistical analysis

SPSS version 17.0 (SPSS Inc., Chicago, IL, USA) was used for statistical analyses. The Shapiro-Wilk test was used to determine whether the variables were distributed normally. Categorical variables were presented as frequencies and/or percentages, and continuous, normally distributed variables were stated as mean ± SD. Student’s t-test or the Mann-Whitney U test were performed to compare normally distributed continuous numeric variables, and the Chi-square test was used to compare categorical variables between the two groups. In order to determine the sensitivity and specificity of PLR values to predict PPROM, receiver-operator curve (ROC) analysis was performed. The area under the curve (AUC) value, specificity, sensitivity were reported. A p value of ≤0.05 was considered statistically significant.

## RESULTS

Baseline demographic and clinical features of the patients were shown in Table 1. There was no difference between the two study groups in terms of age, gravida, parity, gestational age and lymphocyte count (p>0.05). The neutrophil count was significantly higher in patients with PPROM as compared with controls (9948.4±3393.2 vs. 7466.1±1698.5/mm³, p<0.001). Similarly, the platelet count was found to be significantly higher in the PPROM group (244.5±60 vs. 210.6±64.8 x1000/mm³, p<0.001). NLR and PLR were both higher in the PPROM group (p<0.001). Correlation analysis revealed that PLR levels were positively correlated with NLR (r= 0.10, p=0.031).

The neonatal outcomes of pregnancies were presented in Table 2. The groups did not significantly differ with regard to birth weight, RDS, APGAR score, and NICU admissions (p>0.05). Sepsis was more common in the PPROM group (34.7% vs. 19.8%, p=0.02).

**Figure 1:**
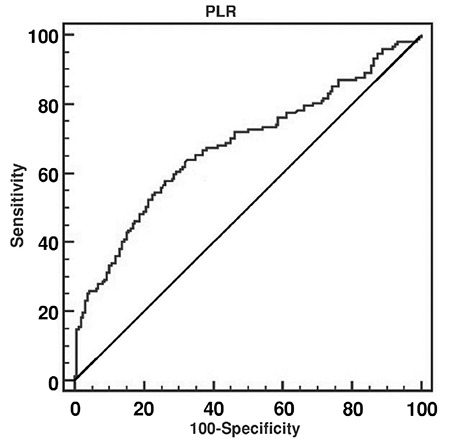
Receiver operating curve for PLR for the diagnosis of PPROM *PLR: platelet to lymphocyte ratio, PPROM: preterm premature rupture of membranes*

The ability of the PLR to diagnose PPROM was evaluated using ROC curve analysis. The AUC for PLR was 0.62 (p<0.001) (Figure 1). The sensitivity and specificity of the PLR was 57.8% and 73.7%, respectively, at a threshold >117.14. PLR values >117.14 were significantly related with increased risk of PPROM.

## DISCUSSION

The main findings of our study are as follows: (1) NLR and PLR were both significantly higher in the PPROM group as compared with controls (2). With the exception of sepsis, a similar relation was found between the two groups according to the neonatal outcomes of pregnancies; sepsis was more common in the PPROM group (3). PLR values >117.14 were significantly related with an increased risk of PPROM.

PPROM, the exact pathophysiology of which is still controversial, leads to common and serious pregnancy complications such as RDS, intraventricular hemorrhage, necrotizing enterocolitis, sepsis, and sudden intrauterine death due to umbilical cord compression. Recent studies demonstrated that the major etiologic mechanism of PPROM was inflammation (19, 20). However, many inflammatory markers have been studied for their ability to diagnose PPROM accurately; a reliable marker for diagnosing PPROM that can demonstrate intraamniotic or placental inflammation is not evident.

Cytokines that participate in inflammatory reactions have been reported to be associated with PPROM. Satar et al. (21) reported that interleukin (IL)-8 levels were increased in PPROM in maternal serum and in the umbilical cord. Similarly, IL-6 was found elevated only in the umbilical cord, especially in PPROM with microbial invasion and histologic chorioamnionitis (21). In the study of Flídrová and Krejsek (22), cytokines such as tumor necrosis factor (TNF)-α, IL-8, IL-6, and IL-1, were reported to be increased in preterm birth and PPROM.

A study by Popowski et al. (23) demonstrated that C-reactive protein was elevated in patients with PPROM with clinical and histopathologic chorioamnionitis. Also, procalcitonin, proadrenomedullin, and serum amyloid A levels were reported to be related with chorioamnionitis before any clinical signs appear (24).

Another marker that can play a role in inflammatory processes is NLR. In systemic inflammatory conditions, leukocyte subtypes differentiate as an immune response. Neutrophil counts increase and lymphocyte counts decrease. As such, the NLR tends to alter in various systemic inflammatory diseases. Several studies demonstrated the prognostic and predictive value of increased NLR in cancers such as colorectal cancer, lung cancer, and hepatocellular carcinoma (25, 26, 27). Also, NLR was found significantly altered in many conditions of pregnancy. Kurtoglu et al. (28) reported high NLR values in preeclampsia. Likewise, NLR values were found altered in gestational diabetes, intrahepatic cholestasis, hyperemesis gravidarum, and acute appendicitis of pregnancy (15, 16, 29, 30). In the study of Köseoğlu et al. (24), NLR was higher in the PPROM group than in controls. The authors concluded that NLR was a useful marker for predicting PPROM (24). In our study, we found higher NLR levels in the PPROM group, consistent with the literature.

PLR is a widely-used marker, which has been demonstrated to predict thrombotic events, inflammatory diseases, and malignancies. Many previous studies reported a significant association between increased PLR and major adverse outcomes in cardiovascular diseases, and reduced survival in malignancies such as pancreatic, colorectal cancer, and endometrial cancer (13, 14, 31, 32). In pregnant women, PLR was investigated in gestational diabetes, acute pancreatitis, preeclampsia, and PPROM (10, 15, 17, 33, 34). In PPROM, Ekin et al. (10) found that the PLR showed no significant alteration between their oligohydramnios and normal amniotic fluid index groups. Another study that investigated the relationship between PLR and PPROM was designed regarding the latency period. PLR was not found to be significant between latency periods <72 hours and >72 hours (34). In our study, regardless of the latency period and amniotic fluid index, statistically significant PLR values were found in patients with PPROM.

In conclusion, we demonstrated an important relation between PLR values of more than 117.14 and the occurrence of PPROM. Moreover, it was found to be a significant independent discriminator for PPROM, a condition that leads to adverse maternal and neonatal events. PLR is a cost effective, easy to use, and practical marker that can be used for the early diagnosis of PPROM, which can help to provide maternal and fetal well-being.

### Study limitations

There are some limitations of the present study. First, this study has a small sample size. Second, it lacks the measurement and correlation analysis of well-known inflammatory markers such as C-reactive protein.

## Figures and Tables

**Table 1:**
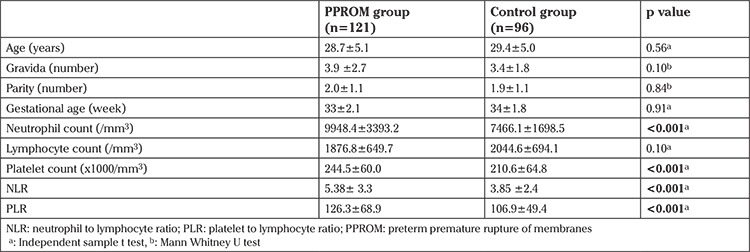
Demographic and clinical characteristics of patients

**Table 2:**
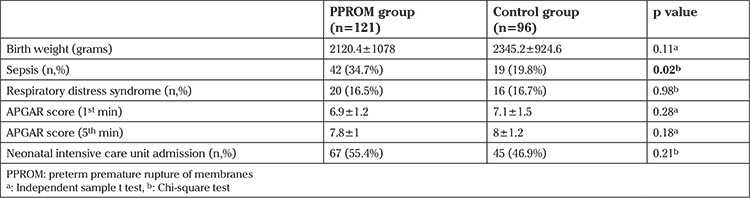
Neonatal outcomes of pregnancies
